# Determination and quantification of PCBs, POCs and PAHs in *Thunnus thynnus* from the Straits of Messina (Italy)

**DOI:** 10.1016/j.dib.2016.02.027

**Published:** 2016-02-15

**Authors:** Emanuele Saija, Valentina Mangano, Katia Ermina Casale, Giovanna Loredana La Torre, Giacomo Dugo, Andrea Salvo

**Affiliations:** Dipartimento di Scienze dell׳Ambiente, della Sicurezza, del Territorio, degli Alimenti e della Salute (S.A.S.T.A.S.), University of Messina (Italy), V.le F. Stagno d’Alcontres 31, 98166 Messina, Italy

**Keywords:** Fish, Thunnus thynnus, POPs, PCBs, PAHs, HRGC-MS/MS

## Abstract

This data set is composed to assess the accumulation of persistent organic pollutants (POPs) such as polychlorinated biphenyls (PCBs) dioxin like (DL) and not dioxin like (NDL), organochlorine pesticides (POCs) and polycyclic aromatic hydrocarbons (PAHs) in *Thunnus thynnus* and to elucidate the suitability of this species as a bioindicator for monitoring contaminations of these compounds in the marine ecosystems of the Straits of Messina.

This investigation was conducted on liver samples of 14 *T. thynnus* collected during April 2015. Quantitative determination of PCBs (DL and NDL), POCs and PAHs in the examined samples has been carried out by HRGC-MS/MS.

Among the PCBs, high prevalence of DL was found while, generally, the values detected for PCBs–NDL were lower than the legal limits. Tuna samples analyzed for PAHs residues revealed that all the samples were contaminated with acenaphthalene, fluorene, phenanthrene and anthracene. Moreover, generally the residual levels of DDT and DDT metabolites were low.

The total content of PCB–DL, in almost all the samples, showed higher concentration than the legal limit.

## **Specifications table**

**Table:** 

Subject area	*Toxicology*
More specific subject area	*Food science, contaminants, biomagnification*
Type of data	*Table*
Data format	*Analyzed output data*
Experimental factors	*POPs determination was performed on tuna tissue (liver samples) that were homogenate before extraction.*
Experimental features	*POPs residues were analyzed after clean-up by HRGC-MS/MS*
Sample source location	*Messina, Italy*

## Value of the data

• The multiresidual analytical method, elaborated in our laboratories for other food matrices, has proven effective and reliable for the determination of many POPs in tuna samples.
• The use of the MRM technique allows, in a single chromatographic analysis, the simultaneous determination of several POPs with different chemical characteristics.
• Data on the levels of POPs is useful to assess quality and safety of the analyzed samples.
• This food must be controlled in order to define the manner in which the sources of environmental pollution can increase the level of contamination.

## 1 Data and experimental design

One of the focal points of food safety is the presence of chemical contaminants. Among the different sources of contamination which food such as fish is exposed, you can include those due to some of the most persistent organic pollutants, such as organochlorine pesticides (POCs) still present in the soil and river sediments as a result of their uncontrolled use in past years, polychlorinated biphenyls (PCBs) resulting from industrial emissions [1], [2], [3], [4] and polycyclic aromatic hydrocarbons (PAHs) that are also distributed in colloidal dispersion of aqueous environment [5].

Many toxic effects on the reproduction, development and immunological function of animals from the use of POCs have been reported. POCs such as aldrin, chlordane (CHL), dieldrin, endrin, heptachlor, dichlorodiphenyltrichloroethane (DDT), hexachlorobenzene (HCB), mirex and toxaphene were listed in the initial “dirty dozen” agents in the Stockholm Convention in 2001. In 2009, hexachlorocyclohexane (HCH) isomers such as α-HCH, β-HCH and γ-HCH were added to the list of persistent organic pollutants (POCs) of the Stockholm Convention for their potential adverse effects on humans and ecosystems [6]. Now, most nations and regions have restricted or banned the use of persistent POCs.

Among the 209 possible PCB congeners, only 12 compounds (PCBs n. 77, 81, 105, 114, 118, 123, 126, 156, 157, 167, 169 and 189) have a degree of toxicity comparable to that of dioxins [7]. The monitoring of PCBs contamination in food, moreover, is also based on the determination of seven congeners target (PCB congeners n. 28, 52, 101, 118, 138, 153 and 180) and CONTAM (Scientific Panel on Contaminants in the food chain of EFSA, European Food Safety Authority) has set represents approximately 50% of all NDL–PCB in food [8].

Many researches on polycyclic aromatic hydrocarbons (PAHs) established the mutagenic and carcinogenic effects from chronic exposure to PAHs and metabolites [9], [10], [11]. Particularly, the European Food Safety Agency (EFSA) in an opinion article released in 2008, pointed that the carcinogenicity of PAHs through oral administration is the most relevant and that in particular, benzo(a)pyrene, chrysene, benzo[*b*]fluoranthene, benzo[*k*]fluoranthene, benzo[*a*]anthracene, dibenzo[*a,h*]anthracene are considered as the most suitable indicators of PAH in food [11].

Due to their lipophilic properties, POPs primarily accumulate in fat-rich products exposing consumer of dairy products to significant levels of contamination [12].

Based on this knowledge and considering that POPs measured in animals can directly contribute to detecting, quantifying, and understanding the significance of exposure to chemicals in the environment [13], we aimed to produce reliable and comparable data on the levels of POCs, PAHs and PCBs in tuna samples from the central Mediterranean Sea in order to assess quality and safety levels for consumption.

POPs determination was executed on liver samples of 14 randomly selected *Thunnus thynnus*, after macroscopic observations. Samples of individual fish were removed and frozen at −18 °C until processed for chemical analysis. The levels of individual POCs, PAHs and PCBs were determined by HRGC-MS/MS.

## 2 Material and methods

### 2.1 Fat extraction and clean-up

According to Kalachova et al. [14], tuna liver tissue was homogenate before the extraction. Subsequently, a mixture of distilled water and ethyl acetate with QuEChERS (4:1 magnesium sulfate and sodium chloride) was added to sample in a polypropylene tube. It was shaken vigorously and centrifuged for 5 min at 11,000 rpm. Then, the upper organic phase and the solvent were evaporated using a vacuum rotary evaporator, the fat was recovered and clean-up with a silica glass column. The glass column, packed with glass wool and silica gel was conditioned with *n*-hexane:dichloromethane (3:1). The elution was performed with 30 mL of a mixture of *n*-hexane:dichloromethane (3:1) and finally, the solvent was removed under vacuum at room temperature. Then, the residue was re-dissolved in 1 mL of *n*-hexane containing 50 ng/mL of bromophos-methyl. Each sample was injected into a Shimadzu gas-chromatography mass spectrometer (TQ8030 HRGC-MS/MS Shimadzu) and analyzed with MRM method.

### 2.2 HRGC-MS/MS analysis

The POPS analysis was carried out by HRGC-MS/MS using a Shimadzu TQ8030 system equipped with a ZB-5MS (5% biphenyl–95% methyl polysiloxane) (30 m×0.25 mm; 0.25 μm film thickness) capillary column; the pressure at the head of the column was 29.2 kPa; helium was used as gas carrier at a rate of 30 cm/s and a flow of 0.68 mL/min. The injector was set at 250 °C, in splitless mode for 1 min. The interface temperature was 300 °C. For the analysis the oven temperature was programmed as follows: initial temperature 60 °C for 1 min, from 60 °C to 150 °C at 15 °C/min, from 150 °C to 270 °C at 10 °C/min and then to 300 °C (2 min hold) at 2 °C/min. The electronic impact (EI) source was 70 eV, the acquisition of spectra was performed in Multiple Reaction Monitoring analysis (MRM) using argon as collision gas at the pressure of 200 kPa. All POCs, PAHs and PCBs congeners were identified by their retention times and selected reactions.

The method was evaluated through validation parameters that included linearity, sensitivity, accuracy and repeatability [15].

### 2.3 POPs determination

Table 1 shows the concentrations and standard deviation of PCBs, PAHs and POCs in liver samples of tuna (*T. thynnus*) from the Straits of Messina. The determination of PCBs regarded 18 congeners (PCB–DL: 77, 81, 126, 169, 105, 114, 118, 123, 156, 157, 167 and 189; PCB–NDL: 28, 52, 101, 153, 138 and 180). Among the twelve PCB–DL investigated in tuna samples, it is constantly highlighted the presence of five target congeners (PCB 118, 105, 126, 123, and 157). As regards the PCB–DL contamination, the total content of these pollutants in tuna samples, expressed as TEQ (TEQ=ΣC_i_×TEF), were higher than the legal limits fixed by the EU Regulation [16] (TEQ PCB–DL=0.02 ng g^−1^), with the exception of the sample TT 02. Regarding the six PCBs–NDL researched, the values found in tuna samples were lower than the legal limits (ΣPCB–NDL=200 ng g^−1^) [16], and only the sample TT 03 showed the highest total content of five PCB–NDL (PCB 28, 52, 101, 153 and 180) even if its values were below the law limits.

The POCs standards investigated were α-BCH, β-BCH, γ-BCH, 4,4′-DDE, 2,4′-DDE, 2,4′-DDD, 4,4′-DDD, 2,4′-DDT, 4,4′-DDT. The tuna samples analyzed for POCs residues revealed that all the samples were contaminated with DDT and DDT metabolites; only 2,4′-DDE was detected in two tuna samples (TT 03 and TT 09, respectively). The presence of this metabolite could be correlated to a previous use of DDT in agricultural activity, to high environmental persistence because of their chemical and thermal stability, to different climatic environmental conditions, to marine currents, to different migratory habits of aquatic organisms and to different feeding habits. Generally, the residual levels of DDT metabolites, detected in tuna liver tissues, were low and in accordance with Italian Regulation [17] (Σ DDT=100 ng g^−1^); only sample TT 03 showed that DDT metabolites were at higher levels than legal limits.

Tuna samples analyzed for PAHs residues revealed that all the samples were contaminated with acenaphthalene, fluorene, phenanthrene, anthracene. At the same time, residues of pyrene, benzo[*a*]pyrene, chrysene, benzo[*b*]fluoranthene, benzo[*k*]fluoranthene, benzo[*a*]anthracene, indeno[*1,2,3-cd*]pyrene, dibenzo[*a,h*]anthracene, benzo[*g,h,i*]perylene, that are considered as the most suitable indicators of PAH in food, were lower than their quantification limits.

The regular consumption of fish can make positive contribution to prevention of cardiovascular disease; fish, however, may contribute significantly to intake of hazardous elements from the environment. The determination of POPs, performed on the liver of tuna samples, evidenced that the total concentrations of PCBs–DL were always higher than the legal limits fixed by the EU Regulation [16]; therefore, the ingestion of toxic elements detected in tuna fish samples represents a health risk for the average consumer. Moreover, regarding the data of the other POPs tested, even if their concentration is generally in accordance with the legal limits, nevertheless, it should be kept in mind that regular or excessive consumption of tuna fish might exceed the recommended weekly intake (PTWI) or the benchmark dose lower confidence limit (BMDL_01_), which not necessary exposes a noticeable risk for excessive consumers.

Further research on the quality and safety of tuna fish is necessary to provide more consistent data to safeguard human health and to define the manner in which the sources of environmental pollution can increase the level of contamination.

## Figures and Tables

**Table 1:** Mean concentration (ng/g±SD) of POPs in livers of *Thunnus thynnus*.

**Compounds**	**Samples**										**Law limits** (ng/g)
**TT 01**	**TT 02**	**TT 03**	**TT 05**	**TT 06**	**TT 08**	**TT 09**	**TT 14**	**TT 17**	**TT 20**
**POCS**											
2,4′DDE	n.d.	n.d.	0.64±0.08	n.d.	n.d.	n.d.	1.44±0.34	n.d.	n.d.	n.d.	
4,4′DDE	21.24±4.99	24.3±0.75	122±6.02	14.16±0.40	9.28±0.27	17.05±0.30	10.24±0.41	18.33±0.31	27.67±1.37	30.62±1.80	
2,4′DDD	0.63±0.06	0.92±0.08	1.06±0.14	n.d.	0.38±0.07	0.43±0.10	1.88±0.45	0.67±0.11	0.92±0.50	1.06±0.89	
4,4′DDD	3.28±0.13	4±0.55	5.64±0.18	3.12±0.23	2.14±0.22	2.15±0.23	2.92±0.41	3.84±0.17	5.15±0.47	5.45±1.12	
											
**PAHS**											
Fluorene	n.d.	n.d.	0.24±0.05	0.16±0.09	n.d.	0.38±0.16	n.d.	0.27±0.07	n.d.	0.16±0.04	
Acenaphthylene	n.d.	n.d.	n.d.	n.d.	n.d.	n.d.	0.22±0.10	n.d.	n.d.	n.d.	
Anthracene	n.d.	n.d.	2.46±0.09	3.91±0.21	n.d.	n.d.	1.86±0.27	n.d.	n.d.	n.d.	
Phenanthrene	n.d.	n.d.	n.d.	n.d.	0.38±0.09	1.28±0.21	0.6±0.22	0.73±0.09	0.37±0.08	n.d.	
											
**PCBS**											
PCB 28	0.10±0.03	n.d.	0.53±0.05	n.d.	n.d.	0.17±0.07	0.12±0.05	0.16±0.06	0.15±0.05	0.16±0.04	
PCB 52	n.d.	0.42±0.13	3.43±0.18	n.d.	n.d.	n.d.	n.d.	0.42±0.12	0.5±0.05	0.98±0.21	
PCB 101	3.15±0.14	4.03±0.17	26.44±0.58	2.72±0.09	1.34±0.10	2.81±0.37	2.16±0.34	4.03±0.26	5.45±0.66	5.48±0.78	
PCB 123	0.45±0.04	0.39±0.09	2.04±0.22	n.d.	n.d.	0.42±0.25	0.64±0.25	0.46±0.16	0.56±0.15	0.51±0.07	
PCB 118	2.57±0.29	4.47±0.18	17.5±0.44	3.05±0.08	1.16±0.12	3.37±0.53	4±0.22	4.28±0.37	4.54±0.89	5.66±0.26	
PCB 153	16.11±0.65	19.98±0.64.	98.4±4.8	18.69±0.43	9.05±0.17	24.87±0.62	n.d.	23.52±0.62	29.68±1.22	29.53±0.36	
PCB 138	13.04±0.13	17.29±0.28	4.6±0.44	14.88±0.59	7.58±0.16	20.5±0.85	14.68±0.87	20.49±1.05	25.12±1.26	25.86±0.60	
PCB 105	0.61±0.18	1.08±0.20	4.6±0.76	n.d.	0.42±0.09	0.65±0.22	1.32±0.30	1.14±0.16	1.26±0.50	1.58±0.29	
PCB 126	1.02±0.16	n.d.	5.22±0.26	1.33±0.42	0.59±0.08	2.35±0.45	1.44±0.44	1.34±0.37	2.55±0.91	2.26±0.22	
PCB 167	0.63±0.13	n.d.	3.17±0.11	n.d.	n.d.	n.d.	2±0.32	n.d.	n.d.	n.d.	
PCB 156	0.56±0.11	0.59±0.17	3.24±0.08	0.65±0.18	0.23±0.07	0.88±0.27	1.4±0.27	0.82±0.33	1.11±0.51	1.16±0.33	
PCB 180	7.99±0.63	9.42±0.33	30.02±0.21	8.85±0.52	4.58±0.33	13.84±0.31	11.08±0.39	9.5±1.17	13.36±0.70	13.73±0.35	
PCB 157	n.d.	n.d.	0.59±0.13	n.d.	n.d.	n.d.	n.d.	n.d.	n.d.	n.d.	
											
Σ DDT	25.15	29.22	129.34	17.28	11.8	19.63	16.48	22.84	33.74	37.13	100
TEQ PCB–DL	0.1021	0.0002	0.5226	0.1331	0.0590	0.2351	0.1442	0.1342	0.2552	0.2262	0.02
Σ PCB–NDL	40.39	51.14	163.42	45.14	22.55	62.19	28.04	58.12	74.26	75.74	200

n.d.=not detected.
